# A Matrix Pencil Algorithm Based Multiband Iterative Fusion Imaging Method

**DOI:** 10.1038/srep19440

**Published:** 2016-01-19

**Authors:** Yong Qiang Zou, Xun Zhang Gao, Xiang Li, Yong Xiang Liu

**Affiliations:** 1 School of Electronics Science and Technology, National University of Defense Technology, Changsha, 410073, China; 2 Research Institute of Space Electronics Technology, National University of Defense Technology, Changsha, 410073, China

## Abstract

Multiband signal fusion technique is a practicable and efficient way to improve the range resolution of ISAR image. The classical fusion method estimates the poles of each subband signal by the root-MUSIC method, and some good results were get in several experiments. However, this method is fragile in noise for the proper poles could not easy to get in low signal to noise ratio (SNR). In order to eliminate the influence of noise, this paper propose a matrix pencil algorithm based method to estimate the multiband signal poles. And to deal with mutual incoherent between subband signals, the incoherent parameters (ICP) are predicted through the relation of corresponding poles of each subband. Then, an iterative algorithm which aimed to minimize the 2-norm of signal difference is introduced to reduce signal fusion error. Applications to simulate dada verify that the proposed method get better fusion results at low SNR.

Multiband radar signal fusion (MRSF) technique is an efficient way to get high resolution ISAR image at the state of the art^1^,^2^,^3^. Unlike construct a ultrawide band radar, it expands the echo signal frequency band by filling the gap data at the signal level, which is more flexible and economical. Meanwhile, the MRSF brings some challenges for ISAR imaging algorithms^4^,^5^,^6^,^7^,^8^. Two important questions are multiband signal coherent compensation^9^,^10^ and damped exponential (DE) model parameter estimation^11^.

For the first question, the subband signals are derived from different wideband radars, and the coherent between them cannot be well guaranteed even though high precision synchronization techniques are adopted^12^,^13^. However, high accuracy coherent is crucial for radar imaging, and if lack of compensation, the the target radar images would be defocused and blurring, and it would not be imaged in serious cases. This issue arouse great concern among researchers, and several compensation methods were proposed. Cuomo^11^ in Lincoln laboratory analysed a wide variety of factors which relate to mutual incoherent, and proved that there were a fixed phase and a linear phase (these two factors are the so-called ICP) between two incoherent subband signals. In ref. 11, a modified root-MUSIC and least square algorithm are used to construct DE model of each subband signal. Depending on these models, each subband is extrapolated to get full band signals, then high dimension optimization is applied to find the fixed phase and the linear phase. However, big error would be introduced by the extrapolation when the gap band is wide. In ref. 14, the similarity between high range resolution profile (HRRP) of the subband is utilized to estimate the linear phase, and then a cost function is defined to derive the fixed phase. This method has high calculate efficiency, but the estimation precision is related to the sample number. In refs 9,10, the fixed phase and linear phase were deduced through the corresponding poles expressions which relate to the same scattering centers. Extrapolation error does not exist here, and the calculation burden is also reduced.

For the second question, the DE model is usually transformed to all-pole model and the parameters are estimated by the modified root-MUSIC and least square algorithm^9^,^11^. In ref. 9,11, the poles closest to the unit circle are used to characterize the subband signals, but this principle is not suitable at low SNR cases, for some interference poles may be selected. Once some false poles are chosen, it is hard to avoid big error in poles estimation. Apart from that, Piou^15^ proposed a state-space based method. In his algorithm, a set of matrices that best describe the measured data are determined, and the fitted data are used to interpolate between and extrapolate outside of the measurement bands. And at the meanwhile, he also presented an iterative approach that refines the state-space matrices of the dual band and improves the fitted data in the vacant bands. But this method faced the same problem at low SNR environment.

In order to solve the aforementioned problems, matrix pencil algorithm which has better performance at low SNR is adopted to estimate the poles and its amplitudes. Then the difference between correspond poles of incoherent signals is analyzed, and a high accuracy coherent compensation approach which does not depend on signal extrapolation and sample numbers is proposed. In order to improve the signal fusion precision, an iterative signal fusion process which aimed to minimize the 2-norm of signal difference is introduced. At last, some simulate experiments are carried out to verify the effective of the method.

## Methods

### Multiband Signals Coherent Compensation

In ref. 9, Tian proposed a novel method to estimate the ICP, however, the phase variety caused by the vacant band is not taken into considered, meanwhile the poles estimated by the root-MUSIC method is easily affected by the noise, both of them would result in loss of precision. In this section, a modified method is proposed.

Without loss of generality, considering the fusion of two subbands, ICP are added to the higher subband. For a static target with *M* scattering centers, the baseband echo signal of the lower and higher subbands 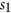, 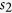 can be written as


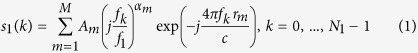



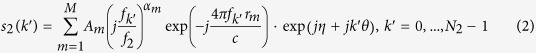


where 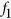, 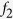 are the start frequencies of the two subbands, 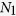, 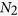 are the step numbers, and the step frequency is 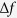, 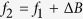, 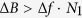. Obviously, Eq. (1) and (2) are the geometrical theory of diffraction model (GTD) expression of 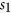, 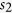, respectively. The model parameters 
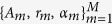
 characterize the scattering centers, and 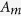 denotes the scattering center amplitude, 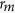 is the relative range of scattering center with respect to the reference point, 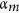 is the frequency depend factor (FDF), which denotes the kind of scattering center. 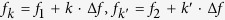, *c* is the velocity of light. 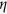, *θ* are the ICP, i.e. the aforementioned fixed phase and linear phase.

Although Eq. (1) and (2) describe the subband signals precisely, it is hard to estimate the parameters accurately due to the FDF. In this paper, the power function 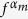 is replaced by the exponential function 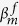, then the GTD model is transformed into DE model, and it can be expressed as all-pole model further (see Eqs (3) and (4)).


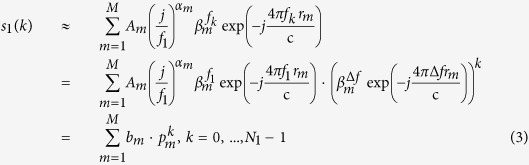



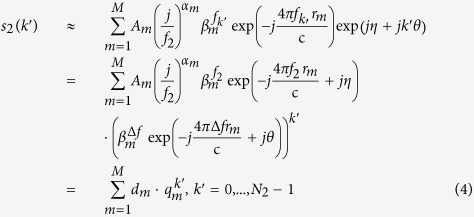


where 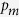 and 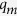 denote the poles of 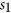, 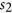, 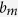, 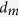 are the corresponding amplitudes.

As a matter of fact, the all-pole model is essentially the summation of harmonic, many harmonic decomposition algorithms can be employed to estimate the parameters, and matrix pencil (MP) is one of the better methods. It was first presented by Y.B.Hua and T.K.Sarkar^16^, and often regarded as one variation of estimating signal parameter via rotational invariance techniques (ESPRIT). It utilizes the properties of exponent signal to estimate the amplitudes and poles simultaneously by solving the generalized eigenvalues of matrix pencil. Through the comparative study of MUSIC, root-MUSIC, ESPRIT, and MP^17^,^18^,^19^, we can draw the conclusion that the MP algorithm has a better performance than the others at low SNR. Then, in this paper, the poles and its amplitudes are solved by MP method.

Taking 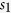 as an example, the Hankel matrix should be constructed first, i.e.


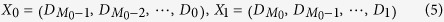


where 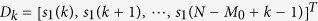, 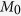 is the pencil parameter, and 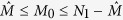. The model order 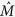 can be obtained by minimum description length (MDL) or Akaike information criterion (AIC) methods, and then the singular value decomposition (SVD) of 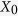 and 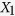 are given by


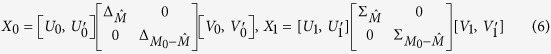


where 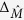, 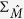 are the diagonal matrix composed by the first 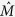 main singular values of 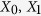, respectively. They and the corresponding matrix 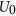, 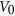, 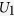, 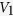 contain the signal information and a little noise information. While 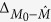, 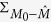 are the diagonal matrix composed by the rest 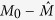 singular values of 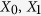, they and the corresponding matrix 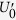, 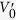, 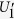, 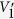 contain noise information only.

If


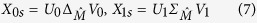


Then the poles 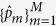 can be estimated by solving the generalized eigenvalues of 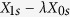, and the amplitude 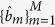 are given by:


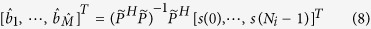


where 
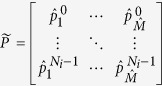
 is a Vandermonde matrix composed by the 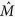 poles.

The poles of 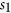, 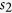 are given below:


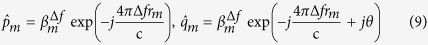


From Eq. (9), it is clearly that the only difference between the same order poles of 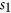 and 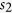 is a linear phase *θ*, and it is given by:


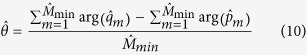


where 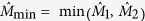, 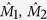 are the estimated poles numbers of each subband, respectively. And 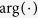 denotes the phase of complex.

Similarly, the amplitudes 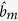, 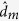 are given by:


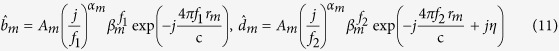


Obviously, the amplitudes are more complex than the poles. Their phases are determined by the fixed phase, the start frequency 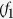, 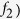, FDF 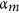 and relative ranges 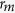, i.e.


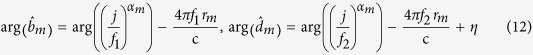


The exact numeric values of 
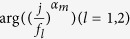
 cannot be given, for 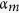 is unknown. Nevertheless, the following relation is satisfied.


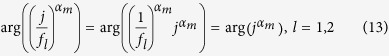


And 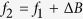, then the fixed phase is given by Eq. (14), i.e.,


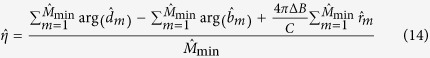


where 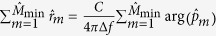


When substituting 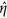, 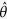 into Eq. (15), the higher subband signal is compensated.


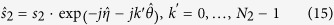


The parameter precision are guaranteed by two aspects:

1. Due to the MP algorithm has good anti-noise performance, the estimate results is interfered slightly and more robust.
2. There is no data extrapolation in this approach, which reduce estimation error.

### Multiband Radar Signal Fusion Imaging

In ref. 9, the band gap is filled by GAPES^20^, and a full band all-pole model is constructed by the modified root-MUSIC, then the fusion images of simulate data are obtained. However, there is no feedback in this algorithm, which is more important for improving the parameter estimation precision. In this section, the full band all-pole model is obtained by MP, and then the gap data is filled by the all-pole model. After that, the initial fusion results are feedback to the previous step until the fusion precision fulfills the requirement.

If 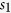, 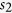 are two subband signals whose length are 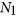, 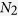, respectively. 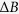 is the band gap between them, and *N* is the samples number of the full band signal. Our fusion approach contains the following steps:

(1) Compensate 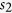 with the aforementioned coherent compensation approach, then we get 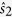 which is coherent with 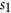.

For the sake of simplicity, let 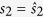, i.e., 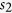 denotes 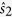 in the following steps.

(2) Construct the full band all-pole model by 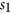, 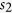. The full band all-pole model is


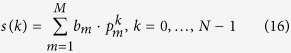


The parameters of Eq. (16) are also estimated by MP, but there are two signals and their frequency band are not overlapped. So the Hankel matrices are constructed a bit differently. Let


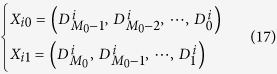


where 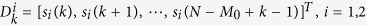.

And the Hankel matrices are


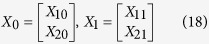


The following parameters estimation process can refer to the previous section.

(3) After the poles and the amplitudes are obtained, the full band signal can be estimated by Eq. (19):


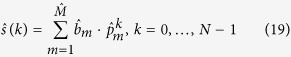


Then the initial estimated subband signals are


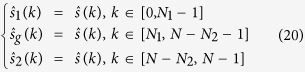


In order to reduce the estimate error, substitute 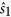, 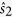 with 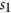, 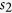, and the initial full band fusion signal is written below:


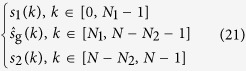


(4) Iterate the fusion result. During each iteration, the approach evaluates the error energy between the estimated signal and primary simulate signal, and the error energy is defined by


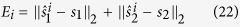


where 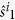 and 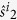 denote the estimated values of the primary simulate signal 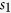 and 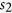, respectively. If 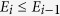, then the algorithm generates a new full band fusion signal based on the 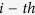 iteration fusion signal. If 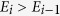, the algorithm stops, and the 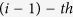 iteration full band fusion signal is just the final result.

**Figure 1:**
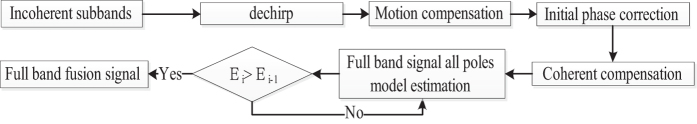
Multiband iterative fusion flow chart.

The flow chart of the proposed algorithm is illustrated Fig. 1, and before coherent compensation several preprocess are added. While this section discuss the processing for only two subbands, it is straightforward to apply this concept to an arbitrary number of subbands.

The full band fusion HRRP can be obtained by apply the pulse compression to the final fusion signal. After that, if we apply pulse compression in cross-range, then the fusion ISAR image is obtained.

## Results

### Model and Simulate Parameter

In order to test the algorithm, the proposed method (A1 for short), the approach in ref. 11 (A2 for short), the approach in ref. 9 (A3 for short), and the approach in ref. 15 (A4 for short) are applied to the GTD model based simulate data and the missile warhead electromagnetic computation model data to fuse multiband signals and obtain the radar ISAR images.

Coherent compensation and HRRP fusion test are carried out on the GTD model based simulated data. And the start frequency 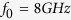, step frequency 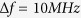, step number 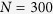, the bandwidth 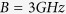 (the theory range resolution is 0.05 m). The target is composed of three scattering centers, the scattering intensity are 2, 1.5, 2.5, respectively. And the FDF and the relative ranges relate to the reference range are −1,1,−0.5 and 0.97,1.05,1.24, respectively. Then the baseband noisy signal is


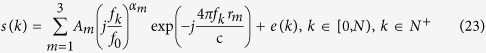


where 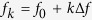, 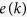 is zero mean white Gauss noise.

**Figure 2:**
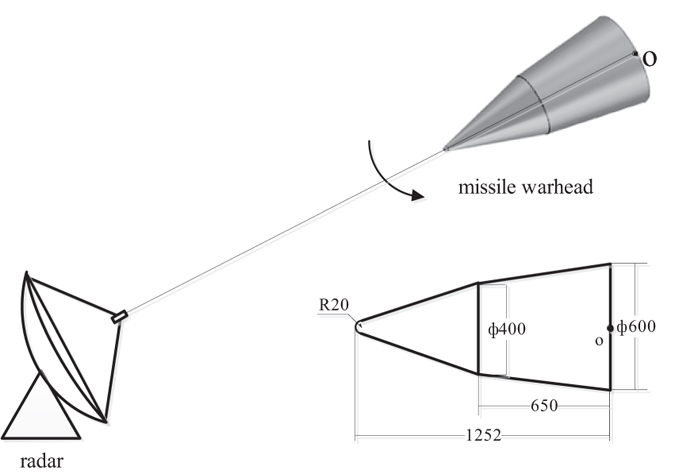
Missile warhead model.

After that, a missile warhead model is constructed in CST2012 environment, and the sweep frequency data of different aspect angle is obtained. This model is a simple missile warhead (Fig. 2). Here the aspect angle is 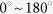 when the warhead rotate counter-clockwise. The parameters are set as follows: aspect angles are 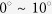 with angle interval 0.1°, frequency range from 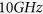 to 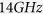 with frequency interval 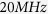. Matrix *X* denotes the electromagnetic computation data, then the number of its column and row are 201 and 101, respectively.

### Multiband Signals Coherent Compensation Experiment

Letting


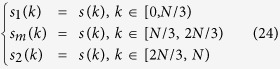


**Figure 3:**
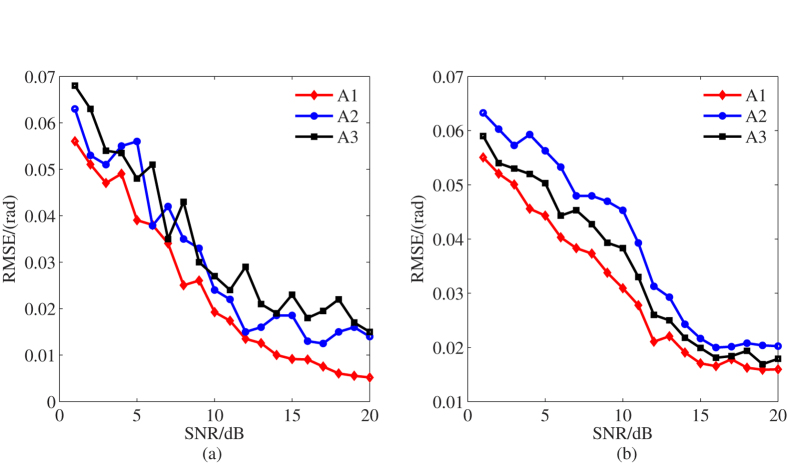
Incoherent parameter estimation result. (**a**) fixed phase estimation result. (**b**) linear phase estimation result.

In order to simulate the effect of mutual incoherence, the higher subband signal 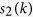 is modulated by 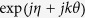, i.e.,


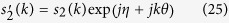


where 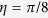, 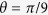. The aforementioned algorithms are applied to estimate 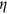 and *θ* at 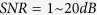
(As approach A4 does not give its coherent compensation method, so this part of test are only carried out on the other three algorithms). Figure 3 illustrates the results of 100 runs Monte Carlo simulation. From Fig. 3, it is obviously that the RMSE of 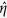 and 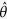 are increased as the *SNR* decrease, and in general, A1 has the lowest RMSE. It is obviously that the proposed method improves the parameter precision at low *SNR*, which verifies the analysis in previous section.

**Figure 4:**
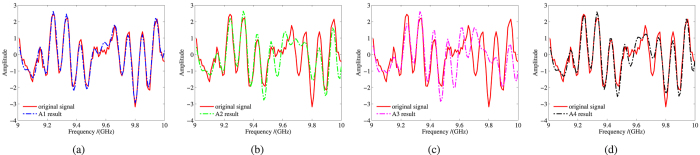
The gap band estimate result. (**a**) the result of A1. (**b**) the result of A2. (**c**) the result of A3. (**d**) the result of A4.

After coherent compensation, 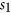, 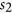 are mutual coherent, the four algorithms are applied to get the gap data at 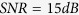. Figure 4 illustrates the estimated gap data 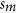 and the primary simulate data. From this figure, all the algorithms can fit the primary data to a certain extent. In general, the result of A1 is better than the other three, and the suboptimal estimated signal is the result of A4. A1 and A4 share one thing in common: they all contain iteration process, and the results show that this process can significantly decrease the fitting error. At the meanwhile, the results also proof the MP method is more efficient than the state-space method in noisy environment.

**Figure 5:**
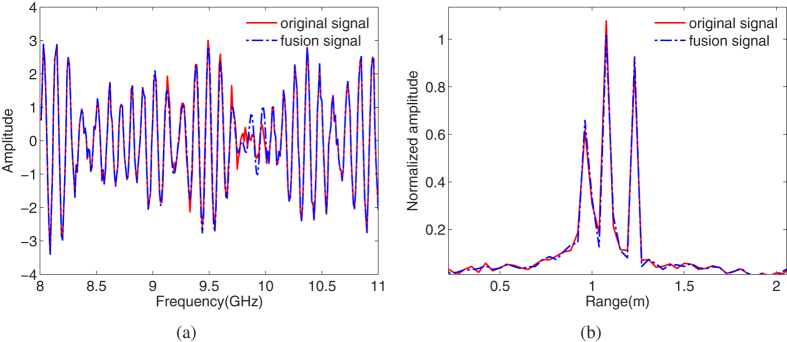
The final full band fusion result of the proposed method. (**a**) real wave. (**b**) HRRP

Denote 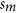 as the estimated data, and the final full band signal 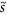 can be derived by 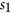, 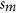, 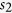 according to Eq. (24). Figure 5 illustrates the real wave and HRRP of 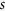 and 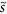. It is hard to recognize the first and the second scattering point in subband HRRPs due to the bandwidth of each subband is 1 GHz, and after the gap data is filled, the full band fusion HRRP can do it easily, which verifies the proposed method improve the range resolution.

### Multiband ISAR Imaging Experiment

Letting


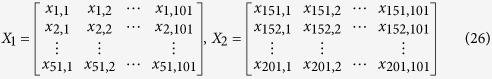


**Figure 6:**
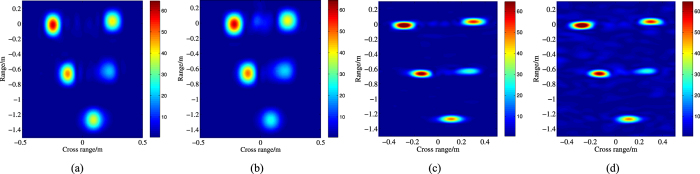
Missile warhead ISAR images. (**a**) lower band 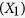; (**b**) higher band 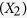; (**a**) full band (SNR = 15 dB); (**b**) full band (SNR = 10 dB).

The frequency band range of 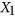 and 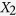 are 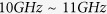 and 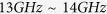, respectively. Figure 6 illustrates the subband ISAR images and the full band ISAR images at two different *SNR*. It is hard to get the precision position of each scattering center due to the poor range resolution of Fig. 6(a,b).

**Figure 7:**
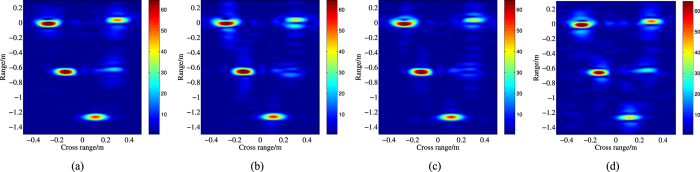
ISAR image of the missile model (SNR = 15 dB). (**a**) the fusion full band ISAR image (A1); (**b**) the fusion full band ISAR image (A2); (**c**) the fusion full band ISAR image (A3); (**d**) the fusion full band ISAR image (A4).

**Figure 8:**
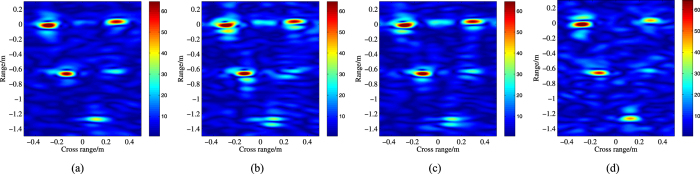
ISAR image of the missile model (SNR = 10 dB). (**a**) the original ISAR image. (**b**) the fusion full band ISAR image (A1); (**c**) the fusion full band ISAR image (A2);(**d**) the fusion full band ISAR image (A3).

Similarly, 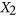 is modulated with the ICP, and 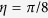, 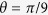. Apply the four algorithms to 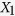 and 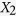 at 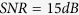 and 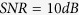, and the fusion results are illustrated in Figs 7 and 8.

Obviously, compared with Fig. 6, the range resolution of the fusion ISAR images in Fig. 7 is improved significantly, and most scattering centers can be positioned precisely. However, in this figure, the fusion images are defocused at different level which result that some scattering points become blurred, such as the second point in the middle of Fig. 7(c). And for Fig. 7(b), there are strong shadows around some points. Owe to the iteration process, Fig. 7(a,d) have the better focused effect, but the shadow of Fig. 7(d) is a little more than Fig. 7(a). Comparatively speaking, Fig. 7(a) is more similar to the original ISAR image (Fig. 6(c)) than the other three.

As the *SNR* decrease to 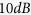, the original noisy ISAR image (Fig. 6(d)) is still clear, but the quality of the fusion image is degraded seriously. Although the range resolution of the fusion images in Fig. 8 is improved, a lot of strong false scattering centers emerge. For Fig. 8(b,c), it is hard to distinguish some false scattering centers from the true points. And Fig. 8(a,d) has the similar result, but the latter has more false points. Then it is obviously that Fig. 8(a) is more better than Fig. 8(b–d).

Table 1 list the image entropy^21^ of each ISAR images in Figs 7 and 8. The ISAR image entropy indicates the quality of the image, and the image which has the smaller entropy is better than the larger one. Then from Table 1, we can conclude that the results are consist with the former analysis.

Above mentioned results show that the MP method and the iteration process have better fusion precision than the other three algorithms at the same SNR.

### Band Gap and The Fusion ISAR Images

**Figure 9:**
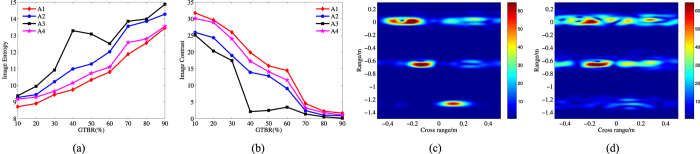
The infection of band gap to ISAR fusion images (SNR = 20 dB). (**a**) the image entropy with different 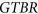. (**b**) the image contrast with different 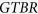; (**c**) the fusion ISAR image of A1 at 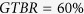; (**d**) the fusion ISAR image of A1 at 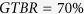.

In this section, the bound of multiband fusion with different subband width and gap width is discussed through the simulated experiments. The missile warhead model electromagnetic computation data is adopted, and all the four algorithms are applied to the subband data which has different gap to total band ratio 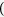
i.e., 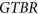, and it defined as 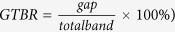 at 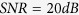. Here, image entropy and image contrast^21^ are used to indicate the quality of the fusion ISAR images, and commonly speaking, the image entropy should as small as possible, while for the image contrast, the larger the better. The results after 100 Monte Carlo simulations are illustrated in Fig. 9. In Fig. 9(a,b), the image entropy increases as the 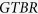 grows, and the image contrast decreases as the 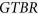 grows, which tell that wide gap bring great difficulties to fusion imaging algorithms. From these two figures, an obvious transformation takes place at 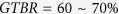
(the results of A3 fluctuate fiercely, but in this range it also has a great change). The fusion ISAR images of A1 at these two 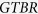 are illustrated in Fig. 9(c,d). Figure 9(d) has serious defocused, and the intensities of some false scattering points even stronger than the true points, so it is hard to recognize the target from it. Relatively speaking, although Fig. 9(c) is defocused, most of the true scattering points can be recognized. If we choose 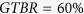 as the critical value for A1, then the other algorithms’ critical values which have the similar image entropy and image contrast are 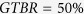
(A2), 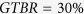
(A3) and 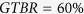
(A4), respectively.

## Discussion

In this paper, MP method is used to estimate the all-pole model parameters which ensure the parameter accuracy at low SNR conditions. After that, two equations are deduced to calculate the incoherent parameters, which can elevate the precision of coherent compensation. What is more, a 2-norm based iterative process is introduced to improve the signal fusion precision. The simulate tests indicate that compared with the other fusion methods, the proposed method can give better fusion signal in low SNR environment. Apart from that, the bound of multiband fusion with different subband width and gap width is also analysed, and the critical values of different algorithms are given in the aforementioned environment. However, although our method has better antinoise capability, it can not work well in colored noise environment, so a modified version of our method should be studied to tackle this problem. At the meanwhile, the present method need a large mount of calculation which impose heavy burden on computers, and it is not suitable for real time process, so in the next step, how to decrease the calculation should be discussed.

## Additional Information

**How to cite this article**: Zou, Y. Q. *et al.* A Matrix Pencil Algorithm Based Multiband Iterative Fusion Imaging Method. *Sci. Rep.* **6**, 19440; doi: 10.1038/srep19440 (2016).

## Figures and Tables

**Table 1:** ISAR Image Entropy.

SNR (dB)	noisy primary signal	A1 results	A2 results	A3 results	A4 results
15	10.3679	11.1461	11.9137	11.8158	11.2824
10	11.2536	12.3927	12.9788	12.7942	12.5729
